# Cooperation and Deception Recruit Different Subsets of the Theory-of-Mind Network

**DOI:** 10.1371/journal.pone.0002023

**Published:** 2008-04-23

**Authors:** Silke Lissek, Sören Peters, Nina Fuchs, Henning Witthaus, Volkmar Nicolas, Martin Tegenthoff, Georg Juckel, Martin Brüne

**Affiliations:** 1 Department of Neurology, Ruhr-University Bochum, BG-Kliniken Bergmannsheil, Bochum, Germany; 2 Department of Psychiatry, Ruhr-University, LWL Hospital Bochum, Bochum, Germany; 3 Department of Radiology, Ruhr-University Bochum, BG-Kliniken Bergmannsheil, Bochum, Germany; Harvard Medical School, United States of America

## Abstract

The term “theory of mind” (ToM) describes an evolved psychological mechanism that is necessary to represent intentions and expectations in social interaction. It is thus involved in determining the proclivity of others to cooperate or defect. While in cooperative settings between two parties the intentions and expectations of the protagonists match, they diverge in deceptive scenarios, in which one protagonist is intentionally manipulated to hold a false belief about the intention of the other. In a functional magnetic resonance imaging paradigm using cartoons showing social interactions (including the outcome of the interaction) between two or three story characters, respectively, we sought to determine those brain areas of the ToM network involved in reasoning about cooperative versus deceptive interactions. Healthy volunteers were asked to reflect upon the protagonists' intentions and expectations in cartoons depicting cooperation, deception or a combination of both, where two characters cooperated to deceive a third. Reasoning about the mental states of the story characters yielded substantial differences in activation patterns: both deception and cooperation activated bilateral temporoparietal junction, parietal and cingulate regions, while deception alone additionally recruited orbitofrontal and medial prefrontal regions. These results indicate an important role for prefrontal cortex in processing a mismatch between a character's intention and another's expectations as required in complex social interactions.

## Introduction

The term “theory of mind” (ToM) describes both the ability to understand and predict the behavior of other people by making inferences about their mental states, their intentions, feelings, expectations, beliefs or knowledge, and to cognitively represent one's own mental states [1]. It is widely acknowledged that ToM evolved in hominids in response to the increasing complexity of social interactions, representing a powerful cognitive tool to determine whether or not a conspecific is willing to cooperate and reciprocate [2], or tends to intentionally deceive and defect at the expense of others [3]. In humans, this cognitive mechanism is more or less permanently “online”, to the extent that we sometimes ascribe mental states to inanimate objects such as cars, computers etc [4]. Given that ToM requires quite large computational resources, it is not surprising that a dysfunction of the ToM mechanism is involved in a variety of neuropsychiatric disorders, including autism and schizophrenia and may cause severely compromised social competence in patients with such conditions [5]–[7].

A number of functional brain imaging studies have revealed that ToM involves an extended neural network located in the frontal, temporal and parietal lobes bilaterally [8], [9]. Specifically, ToM recruits several cortical midline structures, including the medial prefrontal cortex (MPFC), the anterior cingulate (ACC), and the precuneus as well as lateral areas of the middle temporal lobes (MTL), the temporoparietal junction (TPJ), the superior temporal sulcus (STS) and the temporal poles (reviewed in [7], [9]–[11]). The area extending from the anterior cingulate cortex to the anterior frontal pole, particularly the paracingulate cortex, is supposed to be engaged in self-reflection, person perception and in making inferences about others' thoughts [9]. Furthermore, regions near the temporoparietal junction (TPJ) are thought to be involved in reasoning about the contents of another person's mind [12], [13], attribution of a character's actual belief or state of knowledge [11], [14] and the discrimination between self and others [15]. Although hemispheric specialisation has been observed, the results are contradictory: while some studies found selective activity in right TPJ [14], [16], others showed left TPJ to be necessary for representing other persons' beliefs [17], [18]. The mPFC and the ACC are thought to help distinguish self from other, to be engaged in error monitoring, and to differentiate salient from non-salient stimuli [8], [19], [20]. The role of the precuneus is less well known, but this brain area seems to be important for the experience of agency and self-consciousness [21], [22]. The temporal regions around the STS contain mirror neurons that play a decisive role in imitation and learning as well as in recognition of intentional movements [23], [24]. In addition, amygdalar, insular and orbitofrontal activity may contribute the affective “tone” to the evaluation of thoughts and intentions [25]. For example, the insula has been shown to be activated if unfairness is being recognised [26].

A prototypical task used in ToM research has been the ”false belief task”, which requires the subject to predict where a character will look for an object that has been displaced by another character unbeknownst to the first character. While successful performance in this task is considered a milestone in the development of ToM in young children [27], [28], it does not entail “higher order” processes in the framework of interpersonal expectations and intentions such as beliefs of a character about the mental states of a third party–which is crucial to determine whether or not a person has an understanding of the intentions of others (i.e. “ I know that X does not know that Y wants to cheat upon him, and that Y knows that X cannot know what Y really intends to do”).

It is as yet unknown whether an individual's understanding of another person's mental states about cooperative or deceptive intentions of a third party, resulting from false or true interpretations of the third party's actions and behavior, are processed in discrete brain regions of the ToM network. In this study, we therefore sought to examine whether a subject's evaluation of cooperative and deceptive interactions between two or three story characters elicits differential activation patterns within the ToM neural network.

Accordingly, healthy participants were shown cartoon stories depicting scenarios of cooperation, deception or both; the participants' task was to attribute intentions and beliefs to the protagonists. The stories described either a) situations where one person wants another to cooperate to the advantage of both, b) situations where one person deceives another person, and c) situations where two persons cooperate to deceive a third person. Since the outcome of the scenarios was visible to the participants, the experimental design was suitable to examine the test subject's ability to represent a “true” or “false” belief held by one of the story characters. Moreover, the deception condition overtly signalled unfairness, whereas the cooperation condition clearly depicted reciprocity and fairness. By means of fMRI we investigated whether these concepts draw on different brain regions, i.e. whether the representation of a character's erroneous belief in the (unfair) deception condition recruits different brain regions compared to the mental representation of a character's correct inference of intentions in the (reciprocal) cooperation condition.. In addition, the combined cooperation/deception stories were introduced to determine brain regions commonly activated by the formation of a cognitive representation of both a cooperative and deceitful intention. In an additional baseline condition, we showed the same cartoons in jumbled order, the task of the participants was to answer questions regarding physical properties of the stimuli.

Since involvement of temporoparietal junction and precuneus in intention and belief attribution has repeatedly been demonstrated, we expected these regions to be activated across all scenarios. In contrast, we hypothesised that the representation of a scenario depicting a character's concealed deceitful intention would recruit additional brain activation [14]. As a potential candidate for these more complex scenarios we predicted that the medial prefrontal cortex would be more strongly activated due to its involvement in disambiguating information, including discrepancies between one's own expectation and others' (covert) intentions [29]. Moreover, we expected that limbic and orbitofrontal structures such as the insula would differentially be activated by the deceitful scenario, which was associated with a high level of unfairness. We also assumed that increasing complexity of the social interaction in scenarios describing both cooperation and deception and an interaction of three characters would lead to more widespread brain activation due to the higher processing load involved.

## Results

### Imaging

**Figure 1:**
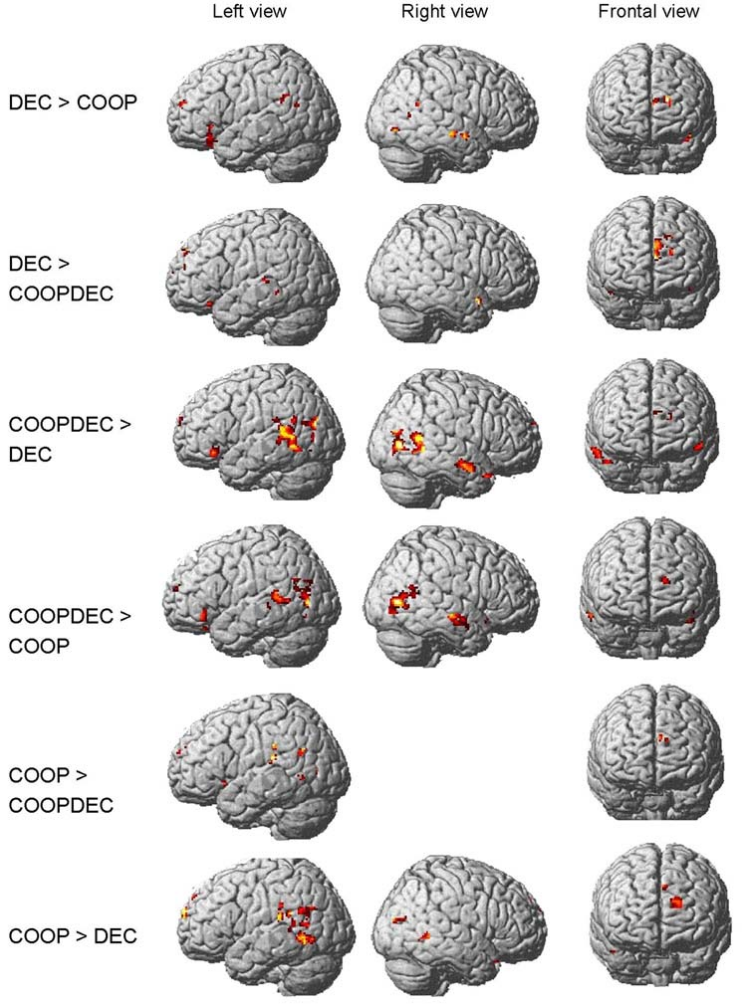
Brain activation in frontal, temporoparietal and temporal regions. Activation patterns are rendered on the brain surface in the contrasts of stories describing deception (DEC), cooperation (COOP) and both (COOPDEC). n = 13, extent threshold k = 15; height threshold p<0.02.

**Figure 2:**
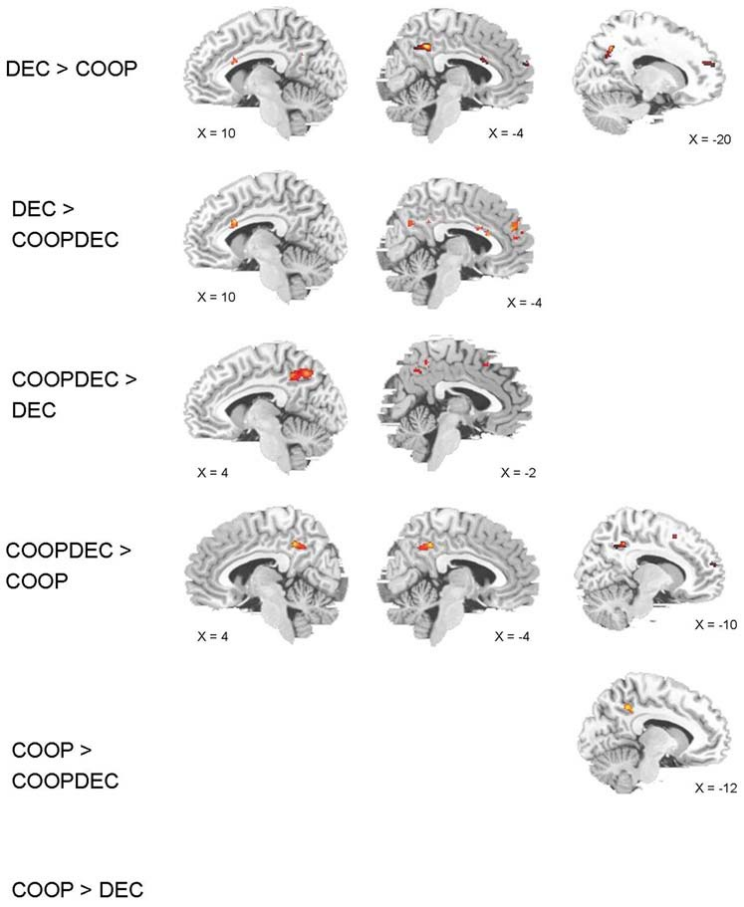
Brain activation in medial frontal, cingulate and parietal regions. Brain activation patterns are shown for the contrasts of stories describing deception (DEC), cooperation (COOP) and both (COOPDEC). n = 13, extent threshold k = 15; height threshold p<0.02.

We analyzed results by directly contrasting all three types of stories, cooperation (COOP), deception (DEC) and cooperation/deception (COOPDEC) with each other, using a height threshold of p<0.02 and an extent threshold of k = 15. The contrasts were calculated for the ROIs derived from the preceding exploratory whole-brain analysis that compared activation during the ToM tasks with activation during the non-ToM tasks. These ROIs encompassed superior, medial and inferior frontal regions, and ACC, insula, as well as parietal and temporal regions including the TPJ and precuneus. In several of these ROIs, mentalizing about stories with a deception element yielded differential activation from mentalizing about stories with a cooperation element. Other regions were commonly activated by both types of stories, with spatially distinct peaks of activation (see Fig. 1 and 2, Table 1).

**Table 1:** Contrasts of the ToM task conditions cooperation (COOP), deception (DEC), and cooperation/deception (COOPDEC) (n = 13; height threshold p<0.02, extent threshold k = 15).

			DEC>COOP				DEC>COOPDEC				COOPDEC>DEC				COOPDEC>COOP				COOP>DEC				COOP>COOPDEC			
*Anatomical region*	*BA*		x	y	z	t-score	x	y	z	t-score	x	y	z	t-score	x	y	z	t-score	x	y	z	t-score	x	y	z	t-score
**Orbitofrontal Cortex**																										
inferior frontal gyrus	47	L	−36	26	−24	3.13					−46	30	−10	4.23	−38	30	−6	3.70								
			−38	28	−8	2.89					−34	20	−16	2.48	−42	30	−22	3.25								
			−42	26	−18	2.77																				
superior frontal gyrus	10	L	−18	48	24	3.11													−16	64	22	4.26				
	9	L					−20	56	40	3.79													−6	56	26	2.82
							−14	50	28	2.35																
medial frontal gyrus	9	L					−8	44	24	4.87																
	10	L	−20	48	14	2.70																				
			−2	58	18	2.72																				
	32	L													−12	12	50	3.97								
**Limbic Areas**																										
anterior cingulate	33	L	−8	14	24	3.48																				
		R					10	18	20	4.28																
	24	R	10	18	24	3.37																				
anterior cingulate gyrus	24	R	2	4	26	2.65																				
	32	L									−10	16	48	7.98												
posterior cingulate gyrus	31	L	−6	−48	38	5.03	−8	−46	32	3.81					−4	−46	42	6.05								
		R	8	−58	30	4.20					10	−44	40	3.76					10	−50	42	5.73				
	23	L					−10	12	28	6.76																
		R					6	14	28	6.73																
posterior cingulate	30	L																					−31	−71	9	3.58
insula	13	L	−40	−46	26	3.69	−32	−36	18	2.93																
	41	L																					−44	−28	14	2.90

			DEC>COOP				DEC>COOPDEC				COOPDEC>DEC				COOPDEC>COOP				COOP>DEC				COOP>COOPDEC			
*Anatomical region*	*BA*		x	y	z	t-score	x	y	z	t-score	x	y	z	t-score	x	y	z	t-score	x	y	z	t-score	x	y	z	t-score
**Temporoparietal Junction**																										
superior temporal gyrus	13	L									−46	−48	16	5.02	−48	−44	16	2.48	−54	−42	22	5.77				
	22	L	−36	−56	18	3.48					−50	−48	8	7.85	−54	−42	8	6.53	−48	−38	4	2.78				
		R	38	−56	16	2.39													−48	−58	16	2.25				
	39	L													−42	−62	28	2.72								
		R									46	−54	10	4.57												
middle temporal gyrus	21	L																	−64	−52	−2	4.44				
	22	L					−56	−42	−4	3.13																
		R									56	−50	−2	3.94					54	−48	−2	4.31				
	37	R									56	−58	12	3.67	42	−58	0	4.85								
	39	L									−40	−76	22	4.33	−46	−70	12	4.14	−46	−58	10	4.21				
											−40	−56	4	3.10									−50	−60	28	3.75
		R	46	−62	10	2.53					52	−74	18	3.60	46	−74	8	4.20	52	−74	18	2.56				
															54	−58	20	2.93								
supramarginal gyrus	40	L																	−58	−52	24	3.11				
**Temporal cortex**																										
superior temporal gyrus	41	L	−40	−44	6	5.43	−38	−42	12	5.25									−52	−32	16	3.36	−38	−32	8	3.72
							−48	−32	8	2.91																
	21	R	52	−4	−12	3.81									60	−12	−16	3.39								
	22	R													52	−18	−8	4.50								
	38	R					46	10	−18	5.20	48	18	−28	2.98												
																										
middle temporal gyrus	21	R	56	−16	−12	4.07					54	−8	−18	3.78												
**Parietal cortex**																										
precuneus	7	L	−16	−62	40	4.44	−12	−68	32	6.22	−2	−56	42	3.09	−2	−66	48	6.12	0	−46	48	3.00				
		R									4	−50	46	7.84	6	−64	46	5.45								
	31	L																					−12	60	26	2.6
postcentral gyrus	40	L																					−58	−32	20	4.74
inferior parietal lobule	40	L																					−64	−32	30	4.53

*x,y,z*: MNI coordinates. BA: Brodmann area. Table denotes the coordinates of local peak activation for the listed brain regions.

As expected, stories containing both cooperation and deception elements recruited the largest regions, in comparison to the other conditions. Specifically, brain activation patterns of the stories containing both elements (COOPDEC) tended to show higher BOLD responses in the majority of ToM-activated regions, i.e. in bilateral TPJ, right anterior temporal cortex, left inferior and superior frontal cortex compared to COOP and DEC, respectively. These results suggest that the processing load for the more complex situation depicted in the COOPDEC scenarios might be higher than for the more straightforward one-to one interactions.

Compared to DEC>COOP, the contrast COOPDEC>COOP showed larger activation in bilateral temporoparietal regions, while both contrasts yielded similar activation in inferior and superior frontal gyrus. Compared to COOP>DEC, the contrast COOPDEC>DEC shows larger activation in inferior frontal gyrus and righthemispheric parietal and temporal regions.

#### Frontal activation

The results from direct contrasts between stories containing elements of deception or cooperation or both showed differential activation patterns. Participants showed higher medial (lefthemispheric BA 9 and 10) and left inferior frontal (BA 47) activation when mentalizing about stories containing an element of deception compared to cooperation. On the other hand, mentalizing about cooperation alone, but not about the combined cooperation/deception stories, led to higher activation in superior frontal gyrus (lefthemispheric BA 9 and 10) when compared to deception alone.

Moreover, within stories containing an element of deception, superior and medial prefrontal activation (left hemisperic BA 9) was higher in DEC compared to COOPDEC, while inferior frontal gyrus activation (lefthemispheric BA 47) was higher in COOPDEC than in DEC.

On the other hand, within stories containing the element of cooperation there was higher activation in left superior frontal gyrus (BA 9) in COOP than in COOPDEC, while the opposite applied in bilateral inferior frontal gyrus (BA 47) and left medial frontal gyrus (BA 32), where COOPDEC stories led to higher activation than COOP stories. A higher activation of COOP in left superior frontal gyrus (BA 10) was also found compared to DEC.

In general, these results suggest that stories containing a deception element activated predominantly left inferior and medial frontal gyrus. In spatially distinct regions of left superior frontal gyrus, higher activation was found in tasks containing only the cooperation element or the deception element, respectively (see Fig. 1 and 2, Table 1).

#### Limbic activation

In left posterior cingulate gyrus (BA 31), both types of stories containing a deception element led to higher activation than stories dealing with cooperation alone, while both types of stories containing a cooperation element led to higher activation in right posterior cingulate gyrus (BA 31) compared to the deception stories.

Further differentiation between stories was found in right ACC (BA 33) and bilateral posterior cingulate gyrus (BA 23), where the activation was higher for DEC than for COOPDEC, and in left posterior cingulate (BA 30), where activation was higher for COOP than for COOPDEC (see Fig. 2, Table 1).

#### Temporoparietal junction activation

Both types of stories containing a deception element led to higher activation in left middle temporal gyrus (BA 39) than cooperation alone, while they activate right middle temporal gyrus (BA 39) less than cooperation alone.

Furthermore, stories containing a cooperation element led to higher activation than DEC in several regions of the TPJ: in left superior and middle temporal gyrus (BA 12, 22, 39) and in right middle temporal gyrus, however, there are no TPJ regions where stories containing a cooperation element commonly show less activation than deception stories (see Fig. 1, Table 1).

#### Other temporal regions

Right middle temporal gyrus (BA 39) exhibits higher activation regarding stories containing a deception element than mere cooperation stories. Within stories containing deception, some regions in right middle temporal gyrus (BA 21, 37) are activated stronger by COOPDEC than by DEC, while a more superior situated region in left middle temporal gyrus appear to be activated stronger by DEC than COOPDEC (BA 22).

There are no temporal regions that commonly exhibit higher activation in stories containing a cooperation element compared to deception. However, some temporal regions show differential activation when comparing COOP and COOPDEC, such as left superior temporal gyrus (BA 41), which is activated stronger by COOP than by COOPDEC, while regions in right superior (BA 21, 22) and middle temporal gyrus (BA 37) are activated stronger by COOPDEC than COOP. In general, stories containing a deception element apparently lead to higher temporal activation than those without. In particular righthemispheric middle temporal regions are predominantly involved in deception processing and/or even more in the combination of deception and cooperation (see Fig 1, Table 1).

#### Parietal regions

Both types of stories containing a deception element yield higher activation in left precuneus (BA 7) than stories with only a cooperation element, however, when comparing deception type stories with each other, this same region is activated higher in DEC than in COOPDEC stories, and also activation in an adjacent precuneus region (BA 31 left) is higher in COOP compared to COOPDEC. No parietal region shows higher activation in COOPDEC compared to DEC and COOP, respectively.

It appears that lefthemispheric precuneus BA 7 is activated predominantly when deception has to be processed, while BA 31 seems rather to be involved in processing cooperation.

### Mean signal intensity

**Figure 3:**
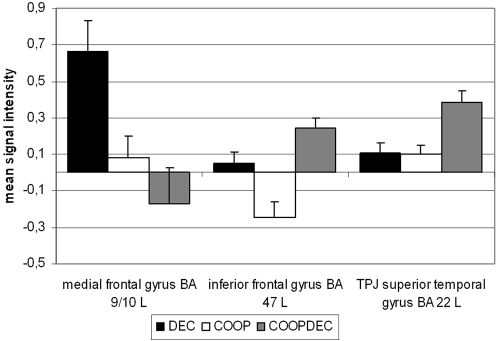
Activation in frontal and temporal regions during the different ToM conditions. The graph shows the mean signal intensity (+/− s.e.m.) (in arbitrary units) in these regions in the conditions DEC (black), COOP (white) and COOPDEC (grey), respectively. The ANOVA with the factors condition and region showed main effects of condition (F(2) = 7.917 p<0.001, region (F(2) = 4.830 p<0.01 and a significant condition*region interaction (F(4) = 9.910 p<0.001 with significantly higher activation in medial prefrontal cortex during DEC compared to COOP and COOPDEC (t(12) = 2.537 p<0.01 and t(12) = 2.290 p<0.01, respectively); in inferior frontal cortex during DEC and COOPDEC compared to COOP (t(12) = 3.041 p<0.01 and t(12) = 4.005 p<0.001), and in temporoparietal junction in COOPDEC compared to DEC (t(12) = 2.079 p<0.01) and COOP (t(12) = 4.173 p<0.001). Activation during DEC was significantly higher in medial PFC than in TPJ (t(12) = 3.179 p<0.01), and during COOP and COOPDEC significantly higher in TPJ than in inferior frontal gyrus (t(12) =  3.726 p<0.01 and t(12) = 2.757 p<0.01).

An ANOVA comparing mean signal intensity in left medial (BA 9/10) and inferior frontal (BA 47) gyrus and in left temporoparietal junction (BA 22) in all three ToM task conditions revealed main effects of condition (F(2) = 7.917 p<0.001, region (F(2) = 4.830 p<0.01 and a significant condition*region interaction (F(4) = 9.910 p<0.001). Paired t-tests comparing activation in the same region for different conditions showed significantly higher activation in medial prefrontal cortex during DEC compared to COOP and COOPDEC (t(12) = 2.537 p<0.01 and t(12) = 2.290 p<0.01, respectively); in inferior frontal cortex during DEC and COOPDEC compared to COOP (t(12) = 3.041 p<0.01 and t(12) = 4.005 p<0.001), and in temporoparietal junction in COOPDEC compared to DEC (t(12) = 2.079 p<0.01) and COOP (t(12) = 4.173 p<0.001). T-tests comparing activation in the same condition for different regions showed significantly higher activation in medial PFC than in TPJ for DEC (t(12) = 3.179 p<0.01), and higher activation in TPJ than in inferior frontal gyrus for COOP and COOPDEC (t(12) =  3.726 p<0.01 and t(12) = 2.757 p<0.01) (see Fig. 3).

### Behavioral measures

Participants performed at ceiling level in the paper-and-pencil ToM story comprehension task that followed the fMRI session. The mean score was 23.0 for answering the ToM questionnaire alone (standard error 0.00) and 59.0 for the ToM questionnaire combined with the sequencing task (standard error 0.00).

## Discussion

In a functional magnetic resonance imaging paradigm using cartoons showing social interactions (including the outcome of the interaction) between two or three story characters, respectively, we sought to determine whether brain areas of the ToM network would be differentially involved depending on the nature and complexity of the observed interaction. The overall activation pattern observed in our ToM task showing activated regions in temporoparietal junction, precuneus, temporal cortex, cingulate areas, and prefrontal cortex corresponds largely to the findings of previous studies and the general notion of the theory-of-mind network [7]–[11]. Since story comprehension of cooperative and deceitful scenarios was flawless in all participants, as indicated by the behavioral data, the observed activations most likely reflect adequate belief reasoning in all three task types. When considering the results from contrasting the three task conditions, it can be assumed that an area that is primarily involved in processing deception will most likely show up in the contrast DEC>COOP, but not its opposite, and potentially also in the contrasts DEC>COOPDEC or COOPDEC>DEC. An area primarily involved in processing cooperation should show up in the contrast COOP>DEC, but not its opposite, and potentially also in COOP>COOPDEC or COOPDEC>COOP.

### Temporoparietal junction, precuneus and posterior cingulate regions are involved in the comprehension of cooperation and deception

Mentalizing about scenarios describing both cooperation and deception (COOPDEC) always showed higher activated areas in the temporoparietal junction when compared to the DEC or COOP conditions alone. Moreover, the opposite contrasts of COOP>DEC and DEC>COOP exhibited activation in TPJ. COOP and COOPDEC tend to activate bilateral TPJ stronger than DEC, with COOPDEC moreover showing higher activation than COOP in these regions. In general, these results correspond to studies reporting temporoparietal activation in ToM tasks requiring belief reasoning [14], [17]. However, in contrast to lateralized effects found in recent imaging studies on belief reasoning, with right TPJ selectively activated in false belief [14] and belief attribution during moral judgments [30], and the findings from lesion studies implicating left TPJ in belief reasoning [17], [18], our results showed bilateral TPJ activation in both cooperation and deception conditions. A possible reason for the disparity of the observed activation pattern compared with previous studies could lie in differences in task requirements. Our stories were designed to force subjects to reason about (cooperative and deceitful) intentions of the story characters, whereas the study by Sommer et al. (2007) used stories where the knowledge of a story character had to be inferred. Hence, the higher processing demands placed on the ToM network by our task may well have recruited more bilaterally distributed TPJ activation than a standard task requiring comprehension of a false belief about the location of an object..

Accordingly, our findings expand upon previous findings on the role of the temporoparietal junction in ToM, suggesting that processing deception, cooperation or both activates bilateral TPJ.

Precuneus activation was also observed in all contrasts of cooperation, deception, and cooperation/deception compared with the other conditions. These findings correspond to the study by Sommer et al. [14], who also found precuneus activation in both false and true belief reasoning about object location. An fMRI study by Ochsner et al. [31] found left precuneus to be one of the regions activated by attributing emotions to other people and the self, together with posterior cingulate and prefrontal cortex. According to Vogeley & Fink [32], the medial parietal cortex-together with medial prefrontal cortex-has a role in taking the first-person perspective and differentiating between actions controlled by the self versus other persons. However, in a PET study by Ruby and Decety [16], there was more bilateral precuneus activation when taking a third person perspective than first person perspective. In an fMRI study that compared thinking about physical causality (physical event and its consequences) versus intentional causality (a subject's intentions and its consequences), the precuneus/posterior cingulate cortex was found to subserve reasoning about intentional causality [33], a function that usually develops before false belief understanding. In accordance with the literature, our results therefore suggest that in ToM tasks, the precuneus performs a rather broad function, relating to perspective taking as well as attribution and processing of emotions and intentions, that is required for belief reasoning including comprehension of cooperation and intentional deception.

Another region commonly activated to varying degrees in all contrasts across conditions is the posterior cingulate gyrus /posterior cingulate (BA 23, 30, 31). Posterior cingulate activation has previously been found in theory of mind research when reading stories about social interaction [34], [35], in particular reading about a protagonist's thoughts [36], and also specifically in tasks focussing on empathy [37]–here together with anterior cingulate, paracingulate gyrus and amygdala. These results hint at a role for posterior cingulate apparently related to social/emotional processing aspects of ToM, which in our study were present in both forms of belief reasoning.

### Processing deception additionally recruits prefrontal cortex, insula and anterior cingulate

Mentalizing about a situation involving intentional deception on the part of the acting character and not recognizing the deceitful intent on the part of the passive character additionally activates left orbitofrontal lateral, inferior, and medial frontal cortex, as seen in the contrasts of DEC vs COOP and COOPDEC, respectively, and in the contrast COOPDEC vs DEC. These results indicate that these prefrontal regions might have a central role in processing a mismatch between intentions and expectations of the protagonists, and also in processing emotional aspects of unfairness [26].

Regions in left lateral superior frontal gyrus, however, showed up in all contrasts, suggesting that adjacent, but spatially distinct areas in this region are involved in processing of cooperation and deception, respectively.

Involvement of different frontal regions in ToM tasks has been observed in previous PET and fMRI imaging studies [15], [34], [35], [38], [39]. These studies, however, did not specify variations of cooperative or deceitful intentions shown in their tasks, nor did they explicitly request to evaluate expectations and intentions of the protagonists in a social setting. These studies revealed either left [34], [35] or right [39] activation of medial frontal and inferior frontal cortex during ToM tasks performance, or right orbitofrontal activation during recognition of mental states [38], as well as specific medial frontal activation [15].

One recent neuroimaging study considering belief processing in ToM associated right lateral rostral prefrontal cortex, but not medial prefrontal cortex, with reasoning about a character's false belief [14]. The authors used a standard false belief task that described hiding and dislocation of objects, which required subjects to predict a behavior without intention attribution–as already pointed out, this constitutes an important difference to our task that may account for differential results.

Two further studies found activation of medial prefrontal cortex in subjects playing games that involved trust and reciprocity, particularly when cooperative intentions had to be evaluated [40], [41]. At first sight these findings might seem contradictory to our results of deception-specific medial frontal activation. However, evaluating cooperative intentions also requires checking for a match or mismatch between one's own expectations and the other's intentions-which might include deception. Therefore such a task may be more similar to our deception task than to our cooperation task, where cooperation was evident and needed no additional evaluation in terms of the truthfulness of the cooperative intent.

Moreover, our results are consistent with lesion studies showing that damage to the medial frontal lobe impaired detection of deception in a ToM task [42] and caused deficits in “affective” theory of mind, including evaluation of another person's emotional situation [43]. It is conceivable that these divergent findings on medial and orbitofrontal involvement in ToM reasoning result from different task paradigms that concentrate either on cognitive or affective aspects of the ToM task. In contrast to classic second-order false belief tasks, which require only a cognitive understanding of the difference between one person's knowledge and that of another, our ToM task required both cognitive and affective ToM in true and false belief conditions. Therefore, the higher activation of medial and orbitofrontal prefrontal regions in tasks requiring both the processing of a malicious intent of one character and the ignorance of that intent by another character might well be related to the stronger emotional valence and perception of unfairness in the deception scenario compared to cooperation.

Interestingly, as shown by Abe et al., orbitofrontal medial PFC has also been found activated when subjects themselves were deceiving another person, [44]. In combination with our findings, these results indicate a general involvement of this region in deception processing, regardless of whether one's own actions or actions of others are concerned. These findings blend in well with the general role suggested for the anterior rostral medial prefrontal cortex (arMFC)–a region which corresponds largely to the area activated in our deception task-by Amodio & Frith [9]. Their review suggests that the arMFC is involved particularly in thinking about mental states and intentions–of self and others.

Orbitofrontal/ventromedial PFC (BA 10/11) and dorsolateral PFC (BA 9/10/46) have also been found to participate in moral judgements [45]–[47]. Before a moral judgment can be made, the inappropriate and harmful intention of an actor has to be detected and linked to empathetic engagement with the deceived person. In our study, participants did not have to judge the moral implications of the scheming person's behavior, because the outcome of each scenario was evident. Thus, subjects were merely requested to describe the deceiver's intention and the victim's ignorance. It is therefore conceivable that the activation during moral judgment in previous studies results from a more complex process in which a malicious intention has to be detected. Inferior frontal gyrus (BA 47) in ventrolateral orbitofrontal cortex has also been found activated in response to moral and social transgressions [48], suggesting that the activation in left BA 47 during deception processing observed in our study may well relate to the moral implications of the depicted events.

However, orbitofrontal cortex activation has to date rarely been found to be involved in theory of mind [23]. It has been suggested that orbitofrontal cortex belongs to a system responding to aversive reactions of others and is therefore also activated in intentional or unintentional violations of social norms [49]. These notions of orbitofrontal cortex involvement in evaluation of moral behavior and violation of social norms loosely correspond to our finding of involvement of orbitofrontal cortex in mentalizing about people who take advantage of the false beliefs of others to transgress social norms.

Bilateral anterior cingulate regions also showed higher activation in conditions containing a deception element, in particular in the contrast DEC>COOP. These results correspond to those by Sommer et al. [14], who found ACC activation in the contrast false vs. true belief. In their comparison of neuronal correlates of ToM and empathy, Völlm et al. [37] found empathy associated with enhanced activations of paracingulate, anterior and posterior cingulate; thus it might be conceivable that higher activation of anterior cingulate regions during processing of false belief situations relates to empathizing with the deceived character. The left insula region (BA 13) also exhibited higher activation in deception compared to the other conditions. In accordance with the results by Sanfey et al. [26], this activation could relate to the perception of unfairness in the deception scenarios.

### Conclusion

Our results suggest that bilateral TPJ, precuneus, and posterior cingulate are regions involved in belief reasoning and evaluation of both cooperative and deceptive intentions of others embedded in a social interaction, at least if the outcome of the social interaction is directly observable. In contrast, orbitofrontal and medial prefrontal cortex, and anterior cingulate regions seem to be predominantly active during processing of a character's ignorance of a malicious intent against him, and attribution of deceptive intentions to a third party. To the best of our knowledge, this study is the first to further dissect the cognitive architecture of processing cooperation versus intentional deception. Our findings provide evidence for the hypothesis that different processes of ToM, namely the comprehension of cooperation and deception, are associated with different activation patterns of the neural network involved in social cognition.

## Methods

### Participants

13 healthy participants (mean age 26.46 years, SD 5.3 years, range 22–38 years; 4 male participants, mean age 26.25 years, SD 4.78; 9 female participants, mean age 26.55 years, SD 5.79) without a history of neurological or psychiatric disorder or first-degree relatives with such illnesses took part in this study after giving written informed consent. The protocol was approved by the local ethics committee of the Ruhr-University Bochum. Prior to the experiment, participants received a handout informing them about the MRI procedure and the instructions for the ToM task.

### Theory of Mind Task

**Figure 4:**
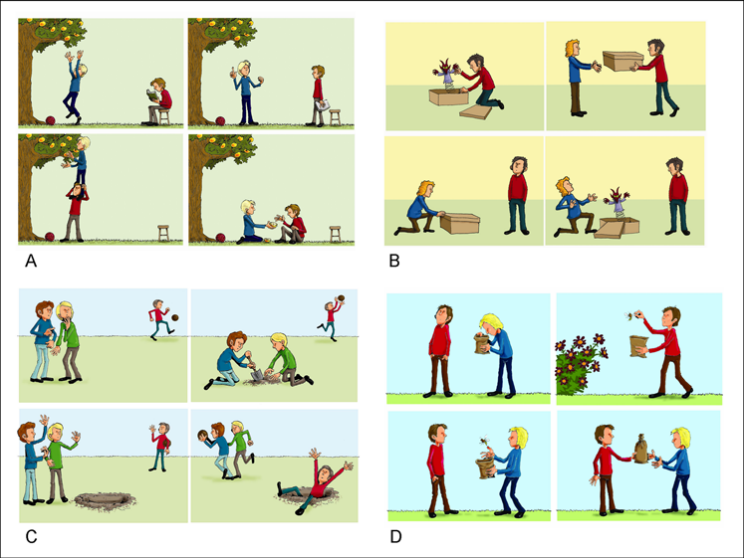
Examples of the ToM cartoon stories presented to the subjects. Panels show (A) cooperation, (B) deception, and (C) cooperation/deception. (D) shows an example of a jumbled cartoon story presented in the non-ToM condition.

The theory of mind (ToM) task consisted of six different cartoon stories with four pictures each [50], showing scenarios of: a) cooperation of two persons depicting reciprocality, b) deception, where one person deceives another person associated with overt unfairness, and c) cooperation of two persons to the disadvantage of a third person,-i.e. two cartoon stories of each type (Examples see Fig. 4). In order to compare activation elicited by ToM demands with non-ToM activation, we introduced a control (non-ToM) condition, where the pictures of the stories were presented in jumbled order.

For the purpose of acquiring fMRI data during performance of the task, the cartoon stories were projected onto a screen during the MR scanning session and presented to the participant via a 45° angled mirror fixed on the head coil. The mirror was adjusted to enable each participant to view the screen without having to move the head. Prior to scanning, a test image was displayed on the screen to ensure that the images were in focus and that the participant could comfortably see the pictures and read the questions. All four pictures of a given story were shown simultaneously on the screen, arranged in two rows in left to right order. In each condition (cooperation, deception, cooperation/deception and non-ToM control), at first the cartoon story was presented alone for 15 sec, then two questions were successively superimposed upon the screen (between the first and the second row of pictures) for 12 seconds each. The task of the participant was to regard the story attentively during the first phase and to think about the answer to each question as long as the question was displayed on the screen.

In the ToM conditions, the questions referred to intentions and beliefs of the protagonists. While one question always referred to the intention of the acting character(s) (e.g. “What does the boy with the red pullover have in mind?”), which could be positive (cooperation) or negative (deception) for the other; the second question pertained to the belief of the reacting character (e.g. “What does the boy in the blue pullover expect from the boy in the red pullover?”), which could be false or true. False beliefs included the incorrect assumption that the other person wanted a positive social interaction (to cooperate, to play, to give a present) or had a problem and needed help. True beliefs correctly assumed a desire for a cooperative social interaction. In the non-ToM control condition, the questions referred to properties of objects displayed on the scene (e.g., “Is the background blue or yellow?”).

The cartoon stories for the ToM and non-ToM condition were presented alternatingly in a blocked design with a total of 12 phases (6 ToM phases and 6 non-ToM control phases) of 39 sec duration each, always beginning with a non-ToM phase, with conditions of cooperation, deception and cooperation/deception presented in randomized order. Each experimental scanning session had a duration of approx. 7 min 48 secs.

### Behavioural measures

After the scanning procedure, the participants completed a paper and pencil version of the ToM task. In the first part of this task, the four pictures of each story were presented in a jumbled order and participants had to put them into the correct sequence. For each cartoon story sequenced correctly, subjects received 6 points (max. score 36 points). In addition, 23 open questions pertaining to the mental states of the cartoon characters were given, i.e. the 12 questions from the scanning session plus additional questions. Here each correct answer scored 1 point (max. total 23 points). The maximum total score for sequencing and questionnaire was 59 points (for details, see [51]).

### fMRI Data Acquisition

Data were acquired using a whole body 1.5 T scanner (Magnetom Symphony, Siemens, Germany) equipped with a high power gradient system (30 mT/m/s; SR 125 T/m/s), using a standard imaging head coil. Blood-oxygen level dependent (BOLD) images were obtained with a single-shot SpinEcho-EPI sequence (TR 3000 ms, TE 60 ms, matrix 64×64, field of view 224 mm, slice thickness 3.0 mm, 0.3 mm gap between slices, voxel size 3.5×3.5×3.0 mm). To reduce noise and obtain an adequate signal-to-noise ratio we restrained the subjects' heads in order to prevent head motion, chose a voxel size of 3.5×3.5×3 mm and used a block length of 13 scans ( =  39 seconds) as well as a spatial smoothing algorithm of 6 mm FWHM in the single subject preprocessing. We acquired 30 transaxial slices parallel to the anterior commissure–posterior commissure (AC-PC) line. The area covered by the fMRI scans encompassed the complete cortex area extending from the superior pole of the cortex to the inferior pole of the temporal cortex. Additionally, anatomical images of each subject were acquired using an isotropic T1-3dGE (MPRAGE) sequence (TR 1800 ms, TE 3.87 ms, matrix 256×256, field of view 256 mm, slice thickness 1 mm, no gap, voxel size 1×1×1 mm) with 160 sagittally oriented slices covering the whole brain.

### fMRI Data Analysis

For preprocessing and statistical analysis of the fMRI data, we used the Statistical Parametric Mapping (SPM) Software, Version 5 (Wellcome Department of Cognitive Neurology, London, UK) implemented in Matlab (Mathworks, Sherbon, MA). The first 5 images of each fMRI session (total 157 images), during which the BOLD signal reaches steady state, were discarded from further analysis. Single subject preprocessing consisted of the following steps: realignment for motion correction, normalization to standard stereotaxic coordinates (MNI coordinates), smoothing at 6 mm^3^ voxels, and first-level single subject data analysis. The acceptable limit of head motion was 2 mm for translational movements and 0.5° for rotational movements.

To assess the differences between the individual ToM conditions (i.e. cooperation versus deception, deception versus cooperation/deception and cooperation versus cooperation/deception), we performed second-level paired *t*-test analyses by using first-level contrasts obtained for cooperation, deception and cooperation/deception minus the global non-ToM condition. To do this, in a first-level single subject analysis, contrast images were calculated for activation in the ToM conditions relative to the non ToM condition for each participant. The analysis encompassed the complete presentation phases of the cartoons, i.e. both processing of the story and answering the questions. The individual contrast images were then entered into an exploratory second-level random-effects analysis (one-sample *t*-test) of the activation patterns for all subjects, with a liberal threshold of p<0.02 (uncorrected) and with a minimum cluster size of k = 15 voxels-in order to find the areas involved in mentally answering questions requiring theory of mind in general.

We restricted our further analysis to ToM-relevant areas found significantly activated in this first exploratory analysis comparing all ToM conditions to all non-ToM conditions. These hypothesis-driven regions of interest (ROIs) were identified by extracting activated clusters using the MARSBAR tool [52]. These clusters encompassed significantly activated regions in left TPJ, BA 21,22 (peak voxel at −58 −40 16), left precuneus, BA 7/31 (peak voxel at −6 −54 36), right Insula, BA 13 (peak voxel at 38 −22 24), left anterior cingulate, BA 33 (peak voxel at −2 6 20), right middle temporal gyrus, BA 21/37/39 (peak voxel at 60 −64 6 and 62 −6 16), bilateral inferior frontal gyrus BA 47 (peak voxels at −46 30 −10 and 40 20 −20), left medial frontal gyrus BA 9/10 (peak voxel −2 62 22) and BA 32 (peak voxel −12 16 50) and left superior frontal gyrus, BA 9/10 (peak voxel at −16 53 34). Using the MARSBAR procedure, box-shaped ROIs were refined based on these clusters by applying the end coordinates in the x,y,z dimensions of the activated areas as corner points of the boxes. To compare the different ToM conditions (cooperation, deception, cooperation-deception) with each other, contrasts within the described ROIs were calculated in a first-level single-subject analysis for each of the conditions separately, in each case compared to the overall non-ToM condition, resulting in three basic comparisons per subject. The resulting contrast images were then entered into second-level random-effects group analyses, i.e. into paired *t*-tests, by means of which we calculated direct contrasts between the activation patterns in all three conditions, resulting in six contrasts (DEC vs. COOP; DEC vs. COOPDEC; COOP vx DEC, COOP vs. COOPDEC, COOPDEC vs. DEC, and COOPDEC vs. COOP). Functional imaging results are reported as *t*-scores with a threshold of p<0.02 (uncorrected) and a minimum cluster size of 15 contiguous voxels. In view of the height threshold chosen, a minimum cluster size of 15 voxels was selected in order to further protect against including areas of spurious activation in our analysis. Maxima of significant activation were transformed into Talairach space [53], anatomical labelling was performed using the Talairach Demon database [54].

Mean signal intensities (in arbitrary units) were calculated for all conditions using the MARSBAR toolbox for SPM for several regions of interest that showed activation differences between the task conditions. The resulting mean values for the activated regions were entered in an ANOVA (SPSS 11.5) comparison of the different conditions and regions.
